# Exploratory study into the awareness of heart diseases among Emirati women (UAE) and their health seeking behaviour- a qualitative study

**DOI:** 10.1186/s12905-016-0350-2

**Published:** 2016-11-07

**Authors:** Sarah Khan, Ayesha Khoory, Dhabia Al Zaffin, Meera Al Suwaidi

**Affiliations:** College of Natural and Health Sciences, Zayed University, P.O. Box 19282, Dubai, United Arab Emirates

**Keywords:** Atypical symptoms, Awareness, Health seeking behavior, Heart diseases, United Arab Emirates (UAE), Women

## Abstract

**Background:**

Cardiovascular diseases were the leading cause of death in women in the United Arab Emirates (UAE) in 2010. The UAE is expected to experience a tripling of heart diseases in the next two decades as risk factors for heart diseases increase. Research shows that first year survival rates of younger women suffering from a heart attack are lower than in men. Women present with a wider range of symptoms for heart diseases than men; non-recognition of atypical symptoms may explain the delay in seeking treatment and poor prognosis following heart diseases in women. No known study on awareness of heart diseases among women has been carried out in the Middle Eastern region.

**Methods:**

Social constructionist and interpretivist epistemological approaches have been considered in this qualitative study to explore the awareness of heart diseases and the health seeking behavior of Emirati women. Convenience sampling was used to recruit 41 Emirati women. Three focus groups and six in-depth semi-structured interviews were conducted to obtain data. Thematic content analysis was applied to the data following transcription and translation of recordings.

**Results:**

Emirati women had limited knowledge on heart diseases. Women were generally unaware of the atypical symptoms, commonly experienced by women however they identified most risk factors associated with heart diseases. Lack of awareness of disease severity and symptoms, sociocultural influences and distrust in the healthcare system were considered the main barriers to seeking prompt treatment.

**Conclusions:**

This study clearly identified gaps and inaccuracies in knowledge of heart diseases, which could contribute to delayed health seeking action and possibly poorer prognosis among Emirati women. Absence of initiatives to educate women on cardiovascular diseases in UAE has erroneously deemed it a less serious concern among Emirati women. The findings from this study provide clear indications of the need to increase accountability of the healthcare system and to develop culturally relevant, gender specific, age focused, heart diseases related public health awareness campaigns in UAE.

**Electronic supplementary material:**

The online version of this article (doi:10.1186/s12905-016-0350-2) contains supplementary material, which is available to authorized users.

## Background

Cardiovascular diseases are largely preventable; yet continue to be the leading cause of death globally [1, 2]. In 2012, almost 17.5 million deaths were attributed to cardiovascular diseases of which 7.4 million fatalities were to due to heart diseases [2]. Cardiovascular diseases have commonly been associated with men, however in 2004 cardiovascular diseases accounted for 21 % deaths in men and 23 % in women in Europe [3]. It is the foremost cause of female mortality in USA, killing 40,000 women yearly, almost one death every minute [1]. Statistics from the Ministry of Health of UAE, in 2010, revealed that cardiovascular diseases were the leading cause of death in women in the United Arab Emirates (UAE) [4].

Research has shown that the number of younger women with heart diseases between the ages of 35–44 years is increasing [1, 4, 5]. There is concern that first year survival rates of younger women suffering a heart attack are lower than in men (74 % verses 81 %) [1], with a greater percentage of women dying before reaching the hospital, 52 % women compared to 42 % men [6].

Typical symptoms of heart diseases including chest pain, breathlessness, pain in arms, light headedness and sweating are seen in both genders however, women often present with less recognisable atypical symptoms such as nausea, vomiting, fatigue, back and jaw pain [2, 6, 7]. Two thirds of women dying from heart diseases report no symptoms of heart diseases [1]. Non-recognition of atypical symptoms of heart diseases may explain the delay in seeking treatment and poor prognosis following heart diseases in women [6].

Men are mostly afflicted with myocardial infarction and coronary artery obstruction but women have a higher prevalence of vasospasm and microvascular ischaemia, which could account for differences in presenting symptoms [8, 9]. In his study on 515 women with recent history of acute myocardial infarction, Mcsweeny highlighted that 95 % of the participants experienced prodromal symptoms before the acute attack [10]. Prodromal symptoms including fatigue, sleep disturbances, anxiety, indigestion and shortness of breath were felt up to a month before a severe episode. Early recognition of prodromal symptoms could translate into prompt action and improved prognosis for women [10].

In UK, a study identified structural, socio cultural and personal barriers to uptake of healthcare services for heart diseases [11]. Structural barriers included inadequate transportation facilities and insufficient doctors to handle the healthcare needs of the community. Denial and fear of the disease discouraged people from validating symptoms of heart diseases. Culturally, participants believed they were self-reliant to cope with ailments and were skeptical of the healthcare services [11].

Ruston and Clayton interviewed 83 women admitted to a hospital in UK, despite the presence of at least one risk factor for heart diseases in majority of the participants, most did not perceive themselves at risk for heart diseases [12]. World Health Organisation (WHO) lists unhealthy diet, smoking, alcohol use and physical inactivity as behavioural risk factors for heart diseases, which manifest as hypertension, obesity, raised blood glucose levels and high blood lipids [2]. Women rejected their own risk of heart diseases since they believed lifestyles of men, rather than women contributed to heart diseases. [12].

Studies on middle-aged women in United States of America and Sweden, who had heart diseases or were considered at risk showed that, when confronted by atypical or prodromal symptoms, women preferred self-medication, guidance from friends and family and waited for the symptoms to disappear on their own rather than seek medical help [13, 14]. Inadequate transportation facilities, lack of insurance, geographical distances and responsibilities towards their families prevented women from taking action, unless their symptoms were debilitating [13, 14].

The Middle East region, including UAE is expected to experience a tripling of heart diseases in the next two decades [15]. Prevalence of hypertension is higher in women, 23 % compared to 20.1 % in men [15]. Almost 30.6 % women in the Middle East are obese compared to 16.6 % men [15]. Middle Eastern population has among the highest rate of diabetes globally, while fat and carbohydrate consumption has continued to increase [15].

A qualitative study by Winslow and Honein, 2007 on general healthcare access for Emirati women revealed that decisions related to all aspects of life were influenced by social norms [16]. Women complained of long waiting time at healthcare facilities, poor standards of care, cultural insensitivity and lack of trust in the healthcare system [16]. Affluent Emirati women preferred to go abroad for treatment. There was a heavy influence of folk medicine and advice from family and friends in all aspects of healthcare [16].

There is a dearth of information related to cardiovascular diseases awareness in the UAE. Qualitative and quantitative studies on awareness of symptoms and risks of cardiovascular diseases in the UAE could not be found despite heart diseases being the leading cause of death in women of UAE in 2010 [4]. Poor prognosis following heart diseases in women could be due to delay in seeking treatment. This study seeks to explore awareness of heart diseases and influences on health seeking behavior in Emirati women. Insight gained from this study will be invaluable in promoting health in Emirati women with heart ailments.

## Methods

### Epistemology

The social constructivist epistemology has been used in this study to appreciate the different perceptions on heart diseases, risk factors for heart diseases and approach to healthcare uptake among Emirati women [17]. Social constructionism and interpretivism has been considered to understand how Emirati women interpret severity and risks of heart diseases in UAE, which ultimately impacts their health seeking behavior [17]. The social constructivist approach acknowledges the need to understand sociocultural, historical, political and spatial influences that shape perspectives of people [18].

A qualitative approach using in-depth interviews and focus groups was best suited to explore perspectives that cannot be revealed by quantitative methods [18].

### Setting

The UAE is situated in the Arabian Peninsula and is made up of seven Emirates: Abu Dhabi, Dubai, Sharjah, Ajman, Fujairah, Umm ul Quwain and Ras al Khaimah [19]. Emiratis comprise only 16.6 % of the population of UAE, the remaining population is of expatriates [20]. The UAE ranks 40th on the Human Development Index, has a high per capita income and a Gross Domestic Product of 4 % [20]. A majority, 76 % of the population in UAE is Muslim [20].

### Participants and recruitment

This study was preceded by a cross sectional study on awareness of heart diseases and health seeking behavior in Emirati women, carried out by the principle researcher using a questionnaire. The questionnaire included an option allowing women to choose to participate in further interviews or focus groups on the same subject. Women who agreed to participate provided their contact information. Research assistants screened potential participants and scheduled interviews (Int) and focus group (FG) sessions. Emirati women, above 18 years of age were included in the study. Women were given a participant information sheet to provide information on the study and highlight expectations from the participants before initiating data collection. All participants signed an informed consent form to show willingness to participate in the study and to allow findings from the study to be published.

### Data collection

A total of 41 Emirati women participated in this study. One pilot focus group discussion and interview was conducted after which data were collected through three focus groups and six in-depth semi structured interviews. The focus group discussion guide (Additional file 1) and in-depth interview guide (Additional file 2) have been provided in the supplementary materials section. Following the pilot sessions, questions related to alternative medicine and ‘Hasad’ (evil eye) were added to the interview guide, owing to their cultural relevance to the subject area. The focus groups sessions lasted between 1 h −1 h and 25 min while the interviews ranged from 35 min to an hour. Focus group 1 and 2 were carried out in English in a conference room at Zayed University with 11 and 8 participants respectively, between the ages of 19–21 years. Focus group 3 was conducted in Arabic and included 16 Emirati women, who were all above 45 years of age. Focus group three was conducted in a venue where the participants collected regularly as a group. Interviews where held in venues decided by the participants, to allow them to express themselves in a place they felt comfortable in. Three interviews were carried out in the participants’ homes and the remaining three at their work places. All participants were introduced to the researchers’ credentials before starting off with the focus groups and individual interviews. The primary researcher was identified as an instructor at Zayed University, holding an MBBS and MPH degree, while the co researchers were introduced as Zayed University undergraduate students. Only researchers and participants were present during data collection. The primary researcher carried out the English interviews and focus groups in the presence of the co researchers. The co researchers, who were native Arabic speakers, conducted the Arabic interviews and focus group 3. Data collection details are summarized in Table 1.

**Table 1:** Data collection

Focus group (FG)/Interview (Int.)	Number of participants	Age (years)	Language of interview/focus group	Duration of interview/focus group	Venue
Focus group 1	11	19–20	English	1 h 28 min	Conference room
Focus group 2	8	19–21	English	1 h 39 min	Conference room
Focus group 3	16	45–57	Arabic	1 h 33 min	Regular gathering place for the group
Interview 1	1	27–29	English	35 min	Participant’s private office
Interview 2	1	26–28	English	44 min	Participant’s private office
Interview 3	1	53–55	English	50 min	Participant’s private office
Interview 4	1	52–54	English	32 min	Participant’s house
Interview 5	1	48–50	Arabic	38 min	Participant’s house
Interview 6	1	30–32	Arabic	30 min	Participant’s house

Voice recorders were used to record the discussions; field notes were made during the interviews and the focus groups to add detail to the voice recordings. The recordings were transcribed and Arabic recordings were translated in to English, by professional transcription and translation services after confidentiality agreement was signed. Zayed University ethics committee provided ethical approval to conduct the study (ZU14_041_F). Removing identifying traits and replacing names by numbers maintained participants’ anonymity. The study did not entail questions of sensitive nature, however since women critiqued aspects of governmental healthcare and discussed sociocultural perspectives it was considered prudent to protect their identity.

### Data analysis

Recordings from the English interviews and focus groups were transcribed verbatim while those conducted in Arabic were translated in to English. Each quote in the transcripts was colour coded according to the information displayed. Common codes from all transcripts were compiled in to Microsoft Word documents by the primary researcher and reassessed by the co researchers. Common codes were grouped in to themes and subthemes and Thematic Content Analysis was carried out to identify emerging and recurrent themes [21]. Major themes were discussed along with supporting quotes. Diverse and minor themes have also been highlighted in the results section.

### Methodological considerations

Focus groups are useful in generating explanatory and descriptive qualitative data; this was suited to the exploratory nature of this study [22]. The homogeneity of the focus groups with respect to age and nationality, in a neutral venue, allowed uninhibited sharing of experiences. Such focus group settings dilute the researcher and participant power imbalance that may occur in individual interviews [22, 23]. An active discussion among a group of participants yields richer data compared to the same number of individual interviews [24]. Discussion among such a homogenous group also discouraged exaggeration of views and dominance of views by certain participants [24]. One focus group exceeded the number of ideal participants (6–12 participants) for a focus group since it was a naturally existing group, however as the subject of discussion was not of a sensitive nature the larger number of participants did not have a detrimental effect on the data collection [22].

In-depth, individual interviews allowed for triangulation and deeper understanding of the subject area, which increased the validity and reliability of data obtained through the focus groups, repeat interviews were not carried out [25]. Data collection by female researchers and co researchers of Emirati descent, in a venue chosen by the participants allowed better rapport and reduced power imbalance between the researchers and the participants [26].

Use of reliable, professional transcription and translation services for transcription of all interviews and focus group recordings contributed to the rigour of this qualitative study [27]. Where transcripts were translated from Arabic to English, the co researchers, who were native Arabic speakers and familiar with the local dialect, verified the context and language of the transcript. Transcripts were not returned to participants for further comments [28].

Rigour was further enhanced throughout the study by maintaining transparency, through provision of a clear account of every process of the study to allow transferability to other setting and interpretation by other researchers [25, 29]. Thick descriptions on data, supporting quotes as evidence, prolonged engagement with participants and saturation of views add to the credibility of the research [21, 30]. Adherence to the COREQ guidelines was maintained throughout the study.

## Results

The in-depth interviews and focus groups revealed awareness related to heart diseases among Emirati women and pointed towards factors that influenced their health seeking behaviour. Summary of key findings is displayed in Table 2.

**Table 2:** Summary of key findings

Awareness of heart diseases
• Most women believed diabetes was the greatest health concern and risk factor for heart diseases. • Breast cancer and heart diseases were considered the main cause of death among Emirati women • Other risk factors identified included obesity, smoking, menopause and stress • Women believed left sided chest pain and fainting to be symptoms of heart diseases but were mostly unaware of atypical symptoms of heart diseases, which are more commonly seen in women. • Women generally believed heart attacks were associated with pain unless the episode occurred during sleep
Factors that influence healthcare utilisation
• Lack of awareness of symptoms was considered the main barrier towards taking action for health matters. Women felt they were knowledgeable about breast cancer and diabetes owing to public health campaigns but had not heard much about heart diseases. • Women relied on friends and family for health related information. • Women mainly depended on family members to provide them with immediate attention in a health emergency, many did not know how to call for an ambulance. • Dissatisfaction with healthcare services, lack of accountability of doctors and dearth of experienced, specialised doctors were listed as reasons for opting for healthcare outside of UAE. • Older women showed a preference for female doctors, however generally the gender and religion of the doctor was not a major consideration for Emirati women. Consulting male physicians was culturally acceptable. • Sociocultural influences contributed to delays in seeking health care assistance. Women were inclined to think of their family first and were more likely to consider solutions for ‘hasad’ (evil eye) and household herbs as remedies before seeking professional medical assistance.

### Awareness related to heart diseases

It was evident from the data collected that Emirati women had limited knowledge related to heart diseases.

#### Heart diseases as a health threat

Most women clearly believed diabetes to be the leading health concern in UAE; this concern was trailed by obesity, breast cancer, hypertension and high cholesterol. However when asked to list the main cause of death due to disease in Emirati women, heart diseases and breast cancer were most commonly identified. Perceived vulnerability to breast cancer was evident in all interviews and focus groups possibly due to widespread awareness programmes related to breast cancer in the UAE:

*Because they are a big awareness now in the country about the breast cancer. They encourage women to go for early check-up, because maximum number of deaths for woman is breast cancer in the UAE*. Int 2

Many Emirati women identified heart diseases as a cause of death but the general view reflected heart diseases as a manifestation of other major common health concerns mentioned above, rather than a disease on its own:

*I feel that some diseases develop to cardiovascular, so diabetes, obesity or hypertension or whatever, will affect your heart eventually. And this will cause death*. FG 1

#### Risk factors for heart diseases

Diabetes was considered a major health concern and a risk for heart diseases in Emirati women. Along with diabetes, Emirati women mentioned the following as risk factors for heart diseases:

- Obesity
  Risk factors identified by Emirati women for heart diseases were mostly associated with obesity. This study revealed that woman were concerned about increasing obesity in the Emirates. Emirati traditions, including eating together at gatherings and considering it hospitable to feed guests with lavish meals were highlighted as reasons for increasing obesity in the Emirati population. Upsurge of easily accessible fast food outlets and coffee shops were also recognized as a reason behind growing obesity:
*Like if you go to a gathering or anything you have to eat. If you say no it’s like, no you have to eat! No that’s too little! It’s like forcing you to eat.* FG 1
*The style of life, it’s a luxurious one, less physical, it’s culture part of it; we have lots of visitors, lots of sweets and eating and cooking. And the lifestyle includes food, restaurants and we are in this part of the world where every 3 meters we have a coffee shop*. Int 3
  Exercise is becoming more acceptable for women in UAE than in the past. Gym facilities have become available, yet many women refrain from exercising. Some women are hesitant to exercise in extreme summers while others believe it is unnecessary to exercise unless they are overweight. Exercise is considered a means to reduce weight and to enhance beauty rather than to maintain good health:
*If I go to the gym people everyone would be like, ‘are you here to gain weight? Why are you here? You’re not fat.’* FG 2
*Because they care about their body shape, so this is about beauty. They care about their beauty more than their health.* Int 1
  While obesity remains a concern for Emirati women, it is difficult for them to break through traditions of eating together to celebrate.
- Smoking
  Smoking is not culturally appropriate in the UAE, nevertheless women are held to higher standards of behavioural correctness when it comes to smoking. While in most cases it is acceptable for men to smoke, women are condemned when found smoking. This cultural norm was considered an advantage for women since it reduced their risk for heart diseases. Women clearly associated heart diseases with smoking:
*No, smoking has its disadvantages of course with men more, much more, it’s one of the biggest causes of heart and chest diseases*. Int 5
  Smoking awareness campaigns have increased awareness among men but it was felt that smoking was on the rise among Emirati women. Many Emirati women were believed to smoke secretly:
*Because used to be only male. Now the new generation, lots of female. And this is the new trend. In my generation, no way, no way to find someone smoking, especially woman. It was part of our culture which is ‘ayb’… it was like prohibited, unaccepted! Now the new generation, we are losing it. They are smoking. Male already and, now we add it to the problem that we add female.* Int 3
  There were suggestions that smoking cigarettes was unacceptable in society but there were more leniencies when it came to smoking ‘sheeshah/hookah’ (water pipe), making smoking a definitive risk factor among both men and women in UAE:
*People accept maybe women smoking hookah more that cigarettes*. FG 1
- Menopause
  Almost half of the participants associated increasing risk of heart diseases with menopause yet their explanation for this increase seemed to be aging rather than a consequence of hormonal changes. A minority of younger women identified that female hormones offered some kind of ‘immunity’ to heart diseases. Others felt menopause had no relation to heart diseases:
*I think like woman after menopause (heart diseases) would still be higher, because they’ve lost a lot of minerals, their vitamin. All their body changes, they become weaker, their metabolism is less. And it’s much worse than men*. FG 2
- Other risk factors
  Other risk factors mentioned by Emirati women included mental stress caused by the fast paced life in UAE. Family responsibilities, abusive husbands and lack of respect in homes were also mentioned as a source of stress for women. Some women felt they had more stressful lives compared to men, making them more vulnerable to diseases, however many women felt men led more stressful lives:
*Now everyone is working they have stress, and stress can lead to heart disease*. Int 6
*Nowadays, life pressure, Dubai are very high competitive city. The environment at work, obviously this is major stress. People in very competitive work environment.* Int 3
  Some women identified high cholesterol and hypertension as conditions that could progressively lead to heart diseases. Other less commonly mentioned risk factors included drinking alcohol, chemical preservatives in food and multiple pregnancies in women which weakened the body making it prone to diseases:
*Cholesterol, diabetes affects the heart, obesity has a big impact, stress, psychological pressures as well. FG 1*

#### Symptoms of heart diseases

Chest pain was the most commonly mentioned symptom for heart diseases. Women described the pain to be of strong intensity and mainly on the left hand side of the chest. It was noticed that women in the older age group were more confident of their knowledge on the common symptoms of heart diseases; in most cases these women’s husbands had suffered from acute heart ailments. Women from the older age group mentioned sweating and breathlessness as symptoms more often than the younger age group:

*Breathlessness, as if knives are stabbing you in the chest. For my husband had breathlessness and sweating*. FG3

*It’s like needles* FG 1

Fainting and collapsing was a frequently mentioned symptom, often inspired by images of men crumbling to the floor when hit by a heart attack on television programmes. However confusion between heart attacks and strokes was also seen, since both were associated with collapsing:

*Maybe from the films I anticipated they faint, and they fall down. From the films, they hold their chests*. Int 3

Only a few women documented back pain as a possible symptom of heart diseases. Women were explicitly asked about non-specific symptoms of heart diseases such as pain in the shoulder, jaw, nausea, vomiting, elbow pain, intermittent fatigue, since these symptoms had not surfaced during the discussion. Recognition of non-specific symptoms was minimal and most often these symptoms were attributed to more innocuous ailments rather than heart diseases.

While many women agreed a heart attack could be possible without suffering from symptoms, most felt this could happen only if the attack occurred during sleep. Some women believed a heart attack was a major health event that could not occur silently:

*And there are many people who are sleeping who don’t wake up because they have a heart attack when they sleep*. Int 1

*No. There must be pain, no I think there must be pain.* Int 3

On asking if symptoms of heart diseases in men and women could be different, most women believed symptoms would be the same since it was the same disease spectrum. Some women speculated possible differences due to hormonal disparities among the genders and some felt symptoms were more severe in women. The general opinion among participants suggested a belief that symptoms could vary among individuals but gender difference did not contribute to the difference in symptoms:

*I think the same, because the same disease. I think it’s the same for men and women, but from one to another it’s different*. FG 2

### Factors that influence healthcare uptake

#### Awareness

Lack of awareness was the most commonly mentioned barrier to taking action for health. Women felt awareness on breast cancer and diabetes was on the rise but they had not heard much about cardiovascular diseases. Most awareness regarding heart diseases came from friends, family and community, who personally knew of others suffering from heart diseases:

*It’s mostly about diabetes, cancer, but nothing about heart disease*. Int 2

*Honestly I think if they have it, someone close to them, their mother, their children, they would know. If someone far, no nothing. Because they experienced it. But they wouldn’t know about it if they didn’t have anyone else*. FG 1

Awareness was considered exclusive of formal education, and participants believed even well educated women may not be knowledgeable about heart diseases:

*We have an education but we don’t know more about heart diseases. Because the type of knowledge like the degree you’ve got, it’s not related to the health matters*. Int 2

Since Emirati women failed to recognise the disease and the need for action they were unable to act effectively when required:

*I think it depends on the awareness itself, if I know the symptoms you might go (to the hospital) earlier…because you know them*. FG 1

Lack of awareness, most likely results in women ignoring the prodromal symptoms that are indicative of an impending attack. Women largely opined they would ignore mild to moderate discomfort unless it continued for an extended period of time, in most cases 2 weeks or until the symptoms became severe. Some women would wait for a few hours before seeking medical assistance even when in extreme discomfort:

*We’ll be ignoring it. I mean until it’s unbearable, that’s what we usually do. You just pretend it does not exist. You say it’s going to go away*. FG 2

*But I think I would first wait for a couple of hours or less, then at the end I would call my husband, family, relatives. I’ve never tried contacting 999, so maybe I will*. Int 6

Participants revealed that despite being aware of heart diseases many would not perceive themselves to be at risk and thus would ignore symptoms:

*Yes they are aware but they don’t think it’s important. They think it won’t hit me; it will come to someone else. Yes people don’t really correlate diseases; they think they’re immune to these things. It’s human nature*. FG 2

Action taken once women suspected something was wrong varied, two women said they would encourage coughing, some mentioned the use of aspirin to allay a heart attack, while one woman mentioned carrying out resuscitation:

*Keep coughing and then I would pump your heart in a way to make your heart move. Give you aspirin so you could talk*. FG 3

Women expressed a strong reliance on family members to provide them with immediate attention in a medical emergency. Several women were unsure of how to call for an ambulance, however were aware of the general emergency telephone number (999) that could be used for assistance. Some believed they would drive themselves to the nearest medical facility if a family member were not available to take them:

*Maybe I can call if I’m that bad. If I’m that bad I will call somebody but if I think okay I can drive to the nearest thing. But maybe I’ll drive*. Int 4

Younger women assumed they were more aware of factors associated with heart diseases, however most older women felt they had more exposure to heart diseases thus were more knowledgeable. Some felt older women were more resistant to awareness initiatives and they preferred to maintain their own beliefs:

*Yes, younger have more awareness. Older have beliefs, it’s difficult to change.* FG1

Participants provided multiple suggestions to improve awareness levels in women. Most younger women felt social media including Twitter and Instagram had potentially the widest outreach but they were mindful that social media is an unreliable source of information:

*I think maybe Instagram or WhatsApp would be much better, because maybe they feel closer to the one first thing. But website you don’t know who wrote this one*. FG 1

*WhatsApp videos are terrible, so many false information. And yet so many watch and believe. So there are pros and cons.* FG 2

Some women preferred short videos and advertisements on television and radio. Many believed direct contact from health educators in schools, workplaces and public places would have a stronger impact:

*You choose places where women gather such as associations, centers or schools in order to raise awareness in the next generation.* FG3

*I prefer group to go from house to house, and we are not so many, very minority in this country, to speak with elder people.* Int 3

*We should have seminars, leaflets to be given to households so at least they educate themselves around what to do in this situation if one falls into this situation. Social media, if something is brought to you, like food, if it is brought to you, even if you’re not hungry, you will take some bites. So maybe that one or two lines of a magazine or a leaflet will change lives of many*. Int 4

#### Doctors and healthcare services

Emirati women had a clear preference for specialised doctors while religion; nationality and gender seemed less of a concern while choosing a doctor to consult:

*No, nothing to do with religion. Nothing to do with gender. Nothing to do with nationality. I care about his experience and his image or reputation*. Int 1

Some older women showed a preference for female, Muslim doctors if they had a choice. Despite feeling more comfortable with female doctors many women felt male doctors were more competent than females, thus they would prefer consulting a male doctor.:

*For me I trust males more than females. I don’t know I feel they are, they are just better, more qualified.* FG 1

*If they are good at their job, have experience, should be a woman. When they’re Muslim they will treat you with things that the Lord permitted, maybe for people of other religions they would treat you with things that are forbidden.* FG 3

*To be honest I would prefer a Muslim, but if there wasn’t, it doesn’t matter. The important thing is that he could treat me*. Int 4

There were concerns of insufficient specialised female practitioners and the necessity to consult male doctors in case of complicated health issues. While a cultural preference may exist for female doctors, the need for good quality healthcare made it acceptable and sometimes preferable to consult male physicians:

*I think we still have enough female doctors for general positions, but for specialist they’re mostly male. I feel female comes more because of culture, and male comes more because of quality.* FG1

Women expressed dissatisfaction at the healthcare services provided in UAE, which was reiterated in their eagerness to seek treatment for serious ailments in other countries. Women felt UAE was not at par as yet with other parts of the world in healthcare:

*If it was for something very severe like cancer or something like that, or risky surgery, people tend to go outside. I wouldn’t say more qualified, I’d say they are more advanced.* FG2

*For serious operations it’s always not recommended to do it here. We just started and maybe after 100 years we will be one of the best*. Int 1

There was a lack of trust in the doctors in UAE and many women narrated tales of being misdiagnosed. Frequent misdiagnosis was attributed to lack of accountability of doctors, this contributed to a lack of faith in the healthcare provision by the participants.

*Nobody monitor or check up after the doctors. ‘I’m a doctor, I do whatever I want’. Many people are misdiagnosed.* FG1

Participants preferred visiting private hospitals for their medical needs even though government hospitals provided free treatment. Factors discouraging them from using government facilities included foremost the large crowds and long waiting hours. Other concerns included inability to follow up with the same doctor in the long term:

*Free, but you have to wait for so long, and environment is so noisy. We always like to go for better service. We don’t like to wait for a long queues, no, no*. Int 1

*You can’t find a regular doctor. Once you go to the hospital, today she’s there, tomorrow she’s not there - there’s someone else on duty.* Int 4

Due to widespread insurance coverage, most Emirati women have the option to visit private hospitals, however they were concerned that private hospitals were too commercialized. Many procedures were expensive and not covered by insurance, making some treatment procedures inaccessible to many:

*I think sometimes private they over estimated. They make the thing it looks really big to get money. So they do more MRI’s and so on just to get the money*. FG 2

While women were generally critical of the healthcare services, most believed the emergency services were quite efficient:

*The emergency is very good, and Rashid hospital, one of the best. Really. No, no, no. The delay only with doctors. But in the emergency, no they have very good services, very good services.* Int 3

#### Sociocultural influences

Health- seeking behaviour in Emirati women is heavily swayed by sociocultural influences. Women often delay seeking medical assistance due to a strong sense of responsibility towards their home and families, at the cost of neglecting their own health:

*I think priority, a mum she has a child, children and a lot of responsibility. So I think because of that she make herself in her head the last thing.* FG 2

Most women believed men would be more prompt in seeking medical attention as they were free of social constraints and had a lower pain threshold compared to women:

*Men cannot endure pain they would immediately go to the hospital, but women would postpone going to the hospital.* Int 2

Other factors that possibly explained why women delayed seeking treatment included a stronger faith in ‘hasad’ (evil eye) and in alternative medicine compared to men. ‘Hasad’ is the belief that poor health could be a result of ill will or envy from others:

*Men wouldn’t really think about hasad. But women, they want something to confirm, it’s psychological*. FG 1

While almost all women across all age groups and education levels had a deeply embedded recognition that ‘hasad’ exists, their beliefs regarding ‘hasad’ varied, some felt heart diseases were too serious to be attributed to it. However many felt that even cancer could result from the evil eye. If people believed their symptoms were due to the evil eye, some would resort to spiritual treatment rather than medical assistance, yet most women felt both treatments would go hand in hand.

*The heart is a part of the body, you can have the evil eye put on your eye, on your arm, anything, the human body is one, any part of the body can be affected by the evil eye or become weak,* God knows. FG 3

*Not a doctor. If it’s hasad, this means this person needs to pray. And need to read Quran, pray or go to almutawwa (religious healer) to read Quran, so that you know*. Int 1

*No - you have to take medication also definitely. You cannot just do duaas (prayers) and sit and say ‘Oh Allah, give me this, this, this’ but you’re not doing anything*. Int 4

The desire for women to be treated at home, where their responsibilities lay made alternative medicine using household herbs an attractive option for healthcare:

*Some women believe in this. For example my mother she believe in this, these herbs can cure her in house instead of going to doctor immediately*. Int 2

*I don’t know, women for example would take medicine at home, she is busy with her family, life, house*. Int 5

There was a noticeable lack of trust in allopathic medicines and doctors among Emirati women, making alternative medicine a more appealing option for them:

*I personally stay away from medicine generally, because I’d rather go to the natural routes. If they don’t work I go there (doctors). Because medicine over time, they have side effects.* Int 3

Two women mentioned their faith in the traditional practice of ‘hijama’, cupping which is a practice recommend by their Prophet:

*Cupping (hijama), the Prophet (Peace be upon him) said, ‘My ummah (followers) can be treated in ways, cupping first, drinking honey’, there is a lady (not a doctor) who does cupping, cupping is one of the best things to treat the arteries and things… I mean, we should not overlook these things*. FG 3

Other sociocultural influences mentioned in interviews that could explain delays in seeking medical assistance included the desire to portray perfection. Some Emirati women felt people were wary of exposing their weaknesses, including diseases to public:

*People want to be perfect, they want to show everything is fine and they’re good. So they don’t want to show that they’re bad and sick*. FG 2

Many women in UAE have an obligation or preference to be accompanied by a family member while going to a doctor. Lack of availability of a trusted family member to accompany Emirati women to a doctor may delay accessing healthcare:

*Maybe because of our culture, or maybe because we’re a family and we should be together. And as a lady you can’t go alone, what if something happens to you. But a man it’s ok he can manage*. FG 1

*Usually they go with somebody. Rarely you will find here a woman who will go by herself. Rarely*. Int 1

## Discussion

The Health Belief Model theorises that certain impetuses are necessary for a change in health behaviour in humans [31]. The foremost requirement for a change in health behaviour is the acceptance that a health concern exists (perceived seriousness). This acceptance is not possible without awareness of the magnitude and severity of the health concern [31]. Perceived vulnerability to the disease by individuals follows increased awareness and contributes to action for change in health behaviour. Perceived benefit and perceived barriers are weighed to evaluate the plausibility of making a change. The alteration in behaviour that takes place is a result of knowledge of disease, vulnerability and risks along with consideration of perceived self-efficacy and perceived benefit from making the change [31].

This study clearly identified gaps and inaccuracies in knowledge of heart diseases among Emirati women. Breast cancer was commonly considered the greatest threat to female life, Mosca et al. showed a similar perception among women in US, twice as many women perceived themselves vulnerable to breast cancer as compared to heart diseases whereas 61 % of the women believed cancer was their greatest health concern [32]. These findings are worrisome considering only one tenth of women will get breast cancer compared to cardiovascular diseases [3]. It is estimated that one in two women in the USA will die from heart and blood vessel related disease compared to one in 25 women dying from breast cancer. The perceived vulnerability from breast cancer was higher than that for heart diseases, which could be due to the emphasis given to the health education initiatives on breast cancer in the region. Lack of initiatives to educate women on cardiovascular diseases in UAE has erroneously deemed it a less serious concern for the population, even though participants identified inherent risk factors for heart diseases among Emirati women. Evidence of improvement in awareness of cardiovascular diseases was seen after exposure to health education initiatives in studies by Mosca et al., highlighting the potential value of nationwide cardiovascular health promotion initiatives in UAE [32, 33].

Emirati women believed diabetes was the leading health problem in the region and associated it with obesity. This belief is justified by the high prevalence of diabetes in UAE; almost one in 5 individuals suffer from diabetes [34]. Emirati women linked obesity and its associated diseases with traditional and regional influences. Women reflected on communal gatherings that form part of the Emirati culture; hospitality revolved around feeding guests with fat and carbohydrate laden food, this along with increasing accessibility to fast food outlets were considered responsible for growing obesity. This finding validates claims that fat consumption has risen in the Middle East in the last two decades [15]. Despite concerns of obesity and increasing importance of body image, women in UAE are reluctant to exercise. Hot weather was a common excuse for the sedentary lifestyle of Emirati women despite availability of fully equipped air-conditioned gyms: a finding that was also highlighted by Shara [15]. Berger and Peerson voiced that exercise was inconsistent with social images of femininity and eligibility for marriage in UAE, thus not embraced whole-heartedly [35]. Initiatives aimed to educate Emirati women on preventative healthcare need to acknowledge these cultural norms and find solutions that are acceptable to the local traditions.

Smoking was conventionally considered less prevalent in Emirati women due to the social displeasure associated with female smokers; thus posed an advantage to their health. But most participants felt smoking was on the rise, particularly among young Emirati women, either in alternative forms of smoking such as ‘Shisha’ (water pipe) and ‘Midwakh’ (traditional stick smoking) or while remaining hidden from the society. This revelation is a matter of extreme concern since increased smoking among Emirati women could further escalate the risk of heart diseases in the future. Outreach has to be improved to reach women who smoke under the radar, without exposing them to cultural scorn. Health workers can no longer assume Emirati women are exempt from the risks of smoking.

Contrary to findings by Ruston and Clayton, where stress was attributed to lifestyles and social expectations adopted by men [12], Emirati women felt they had equally stressful lives, even if they chose to be unemployed. Participants felt UAE had a fast paced life where women handled multiple, stressful responsibilities. Efforts to promote mental wellbeing of women in UAE regardless of their employment status could contribute to improvement in overall health of Emirati women.

None of the participants suffered from heart diseases themselves, this was an interesting observation since it meant that the views reflected in this study were perceptions of the common Emirati women population, rather than of cardiac patients, as seen in many other studies [11–14]. As seen in other studies such as by Todd, Read, Lacey and Abbot [32], Emirati woman in this study did not show any cognizance of prodromal symptoms of heart diseases while atypical symptoms of heart diseases were hardly recognized. Non-recognition of common symptoms was cited as a reason for delayed health action in other studies [13, 14]. Typical, media hyped symptoms such as chest pain were generally recognized nevertheless most women expected a heart attack to be an event that could not be missed. This finding is concerning since statistics from American Heart Association show that two thirds of women who die from heart diseases exhibit no obvious symptoms of heart diseases [36]. Efficacy of thrombolytic treatment for an ischaemic heart attack decreases after 6 h of the episode [37]. This makes it vital for Emirati women to recognise possible yet seemingly insignificant warning signs, symptoms and implications of a cardiac event as their first defense against mortality from heart diseases.

Studies have revealed greater awareness pertaining to health in older and more educated women [31], however in this study the younger participants perceived themselves to be more aware of health related issues, there was also a perception that the older generation was reluctant to let go of beliefs handed down to them from their forefathers. The younger participants seemed more adept and keen on using social media, albeit with reservations on authenticity of information relayed for health related information. Most younger women scoffed at the idea of reading brochures and magazines however it seemed evident that older participants, while not averse to social media, preferred more conventional forms of health education, through TV, radio, magazines and newspapers. Several older participants felt they would learn most through personal interaction with health educators. This difference in preferences observed among age groups emphasizes the importance of not only gender specific health education, but also of the need for stratifying health education plans in accordance to the age groups being targeted. Social media and internet can be used effectively by accentuating reliable resources for women to access, yet health education dissemination should not be limited to electronic media.

Participants expressed that most Emirati women were indeed likely to delay seeking medical help. Unless symptoms were severe, women would opt for home remedies first and hope for symptoms to disappear on their own. They were more likely to contact immediate family or friends for assistance rather than call upon an ambulance or go to a doctor. This preference suggests a perception of self reliance among Emirati women, but could also hint towards inability of Emirati women to take major health decisions on their own particularly when it meant placing themselves before their family obligations; similar to findings in other qualitative studies [13, 14]. Herbal remedies were popular among Emirati women and usually the first line of treatment for maladies. Convenience of treatment at home, traditional faith in herbal medicine and apprehension towards doctors and allopathic treatments were cited as reasons for turning towards alternative medicines including ‘Hijama’ (cupping). There is a need for quality healthcare services for women to be available at their home. Community health workers could play a vital role in bringing healthcare closer to women, to prevent mortality due to delayed health access. Healthcare initiatives should acknowledge the social obligations of Emirati women towards their family and find solutions where women are empowered to avail healthcare at the earliest.

Another valuable insight into socio-cultural influences in to Emirati women’s life is seen in their belief that disease can be caused by ‘hasad’, ‘evil eye’. People could intentionally or unintentionally inflict ill health upon others by envying their prosperity. ‘Hasad’ has religious allusions and Emirati women associated any disease that appeared unexpectedly to the ‘evil eye’. Quranic (Holy book) verses and religious scholars were consulted to treat for diseases caused by ‘hasad’. Such beliefs could contribute to delays in seeking medical help, though in most cases women felt they would resort to medical treatment along with spiritual reprieve. Such facets of culture are integral to the life of Emirati women and should be incorporated in to plans for health promotion and education among Emirati women. Allowing provision of spiritual advisors alongside healthcare providers in medical facilities may encourage Emirati women to go to medical facilities earlier.

While social obligations, lack of recognition of symptoms and denial that symptoms could be serious caused delays in seeking medical help; lack of faith in healthcare services seemed a notable concern among Emirati women. As also seen in a previous study Emirati women were discouraged by long waiting times in government hospitals and the inability to follow up with a preferred doctor. Women would rather pay for time-efficient services with a doctor of their own choice than wait hours for free services [16]. While Winslow and Honein unearthed concerns of lack of cultural sensitivity by healthcare providers, Emirati women in this study did not share the same perceptions, however in both studies Emirati women raised concerns of being misdiagnosed [16]. Some women preferred consulting with Muslim, female doctors however the consensus deemed that qualifications of the doctor were of paramount importance. Language was not a major concern as long as participants were able to communicate effectively with the doctors. Women were willing and able to visit male doctors, despite cultural and religious inclinations, if they felt it was in their best interests. A dearth of specialist female doctors was perceived by Emirati women, however this was not seen as a barrier to seeking treatment for most participants. Many women felt men were inherently more skillful as physicians, a possible cultural indication that regards men superior to women. Contrary to other studies, Emirati women did not feel transportation, cost or accessibility to medical facilities formed structural barriers to accessing treatment in UAE [11, 13]. Health insurance and free treatment was readily available to Emirati women should they choose to avail it. These findings reveal that Emirati women are not constrained by culture, language, religion, gender and cost of healthcare providers, but feel strongly about the being treated by competent, trustworthy doctors.

Lack of accountability of health providers has decreased credibility of services and posed a hurdle while seeking healthcare. Majority of Emirati women would seek medical treatment outside the UAE for serious health concerns [16]. This is an important finding since it reveals that women in UAE felt able to place their health needs above other socio-cultural obligations if they recognised the seriousness of their ailment; thus the value for comprehensive health education among Emirati women. It is vital to instill more transparent and stringent monitoring of healthcare service provision in UAE, with repercussions for lapses in quality of healthcare to instill confidence in services. It is imperative that women feel confident in seeking immediate, available treatment as opposed to delaying medical assistance until they can travel to a foreign country.

### Limitations

This study made meaningful contributions on awareness on heart diseases severity, vulnerability, knowledge of risk factors and symptoms among Emirati women. The study gave a deeper insight in to the factors that contributed to health-seeking behavior in Emirati women. We believe including views of expatriate women from UAE in the study could have allowed further in-depth understanding of cultural and religious influences on health seeking behavior among Emirati women.

## Conclusions

This study clearly identified gaps and inaccuracies in knowledge of heart diseases among Emirati women. While public health campaigns on breast cancer and diabetes have seemingly increased awareness and perceived need for action for these disorders, absence of initiatives to educate women on cardiovascular diseases in UAE has erroneously deemed it a less serious concern among Emirati women. The Middle East region, including UAE is expected to experience a tripling of heart diseases in the next two decades. Despite the increasing risks and prevalence of heart diseases there has been very little research on awareness and perceptions of heart diseases in UAE. It is evident from this study that Emirati women lack awareness on heart diseases; their atypical and prodromal symptoms and often delay seeking help for medical concerns. The findings from this study have huge implications on the need for gender specific, heart diseases related public health awareness campaigns in UAE that are tailored to target different age groups. The integral role of friends and family in providing support for health related concerns makes it prudent to consider the role of community health workers in disseminating health education to the community. Initiatives taken to educate Emirati women on healthcare have to allay the mistrust in the healthcare system and acknowledge the cultural norms to find healthcare solutions that are acceptable to the local Emirati population.

## Additional files

Additional file 1: Focus group discussion guide. The focus group guideline that included questions and prompts to facilitate the focus group discussion. (DOCX 106 kb)
Additional file 2: In-depth interview guide. A semi structured interview guide to aide the interviewer during the in-depth interview sessions. (DOCX 77 kb)

## Abbreviations

FG: Focus group
Int.: Interview
UAE: United Arab Emirates
WHO: World Health Organisation
