# Supplementary material for: Exploratory study into the awareness of heart diseases among Emirati women (UAE) and their health seeking behaviour- a qualitative study
Source: BMC Womens Health. 2016 Nov 7;16:71. doi: 10.1186/s12905-016-0350-2 (PMC5100226; doi:10.1186/s12905-016-0350-2)
Supplement: Additional file 1: — Focus group discussion guide. The focus group guideline that included questions and prompts to facilitate the focus group discussion. (DOCX 106 kb) [file 12905_2016_350_MOESM1_ESM.docx]

**Focus group discussion guide**

**Introduction**-

Introduce yourselves, the project. Ask them to fill out details on the name card. Make them comfortable, offer snacks. Let the participants know that there is no right or wrong and their views are valuable to us. Let them know we are recording the session and ask permission to take a picture to make it easy to remember placement later

**Demographics**

**Age, emirate, education level**, **marital status**- to be filled out on name card

**Awareness:**

What is the biggest health problem in in UAE?

- Why do you say so?

What is the major cause of death in women in UAE?

- Why do you say so?
- What is the main source of information on heart health?

If you had nausea, chest/ abdomen pain and fatigue on and off for 1-2 weeks what would you do?

What do you know about heart disease?

What are symptoms of heart attack?

- What about nausea, fatigue, vomiting, elbow pain, jaw pain, back pain etc.

**(Only use as prompts after everyone has exhausted their list of symptoms)**

**If some people mention all symptoms, ask if others knew these symptoms too; which ones did they not know about?**

- Can we have a heart attack without any symptoms?

Do men and women have the same symptoms?

- If different how are they different

**Perceived susceptibility**

Are men and women at the same risk of heart attack?

- Explain
- Smoking?
- Stress?
- Pain threshold? (prompts)

Is their anything in the life of Emirati women that could protect or increase their chances of getting heart disease?

What are the main risk factors for heart disease?

OR

Who is at risk for heart disease?

Is their anything in the life of Emirati women that could protect or increase their chances of getting heart disease?

Does ‘hasad’ play a role in heart disease?

- How would people seek treatment if they believed it was ‘hasad’
- Would they seek medical treatment?

Black magic??

Do you believe we/you could be at risk for heart disease?

**Action**

What would you do if you thought you were having a heart attack?

Do you have a regular doctor you go to?

- Is a regular doctor important to you?
- Why do you prefer that particular doctor?
  - Female doctor- Do we have enough?
  - Nationality
  - Religion?
  - Language?

**Barriers**

Do you believe women delay seeking help for health problems?

- Explain

Is there anything that would prevent you from seeking health care?

- Do you believe that there is a difference between genders when it comes to willingness to seek treatment?
- Why do you believe so?

**Hesitancy to reveal they are ill (cultural? Why?)**

- Cost of treatment in UAE?
- Quality of treatment in UAE?
- Insurance?
- Culture? Permission, family
- Transportation
- Self medication
- Alternative medicine

(Prompts to be used as required)

Would you prefer treatment abroad to UAE?

- Explain
- How satisfied are you with treatment in UAE?

How do you feel about emergency care in UAE?

How aware do you believe Emirati women are of heart disease risks and symptoms?

- How can we increase awareness on heart disease in women?
- Who has an important role to play?

Do you believe awareness on heart disease differs in different emirates?

- Explain
- Age groups
- Educational status
