# Supplementary material for: Exploratory study into the awareness of heart diseases among Emirati women (UAE) and their health seeking behaviour- a qualitative study
Source: BMC Womens Health. 2016 Nov 7;16:71. doi: 10.1186/s12905-016-0350-2 (PMC5100226; doi:10.1186/s12905-016-0350-2)
Supplement: Additional file 2: — In-depth interview guide. A semi structured interview guide to aide the interviewer during the in-depth interview sessions. (DOCX 77 kb) [file 12905_2016_350_MOESM2_ESM.docx]

**In-depth interview guide**

**Introduction**-

- Introduce ourselves and the study
- Explain that there is no right or wrong view
- Their views are valuable to us.
- Inform them we are recording the session
- Ensure consent form has been signed

**Demographics**

- Thank you so much for agreeing to participate
- Could you tell us your approximate age?
- Which Emirate do you live in?
- What is your level of education?
- What have you studied?
- Are you married? Children?
- Do you work?

**Awareness:**

What is the biggest health problem in in UAE?

- Why do you say so?

What is the major cause of death in women in UAE?

- Why do you say so?

If you had nausea, chest/ abdomen pain and fatigue on and off for 1-2 weeks what would you do?

Do you have any form of heart disease?

- If yes- please explain (symptoms, etc.)

Do you know of any Emirati woman with heart disease?

- If yes- explain symptoms, problems

What do you know about heart disease?

- What is the main source of information on heart health?
- What are symptoms of heart attack?
- What about nausea, fatigue, vomiting, elbow pain, jaw pain, back pain etc.

**(Only used as prompts symptoms list has been exhausted)**

- Can we have a heart attack without any symptoms?

Do men and women have the same symptoms?

- If different how are they different

**Perceived susceptibility**

Are men and women at the same risk of heart attack?

- Explain
- Smoking?
- Stress?
- Pain threshold? (prompts)

Is their anything in the life of Emirati women that could protect or increase their chances of getting heart disease?

What are the main risk factors for heart disease?

OR

Who is at risk for heart disease?

Is their anything in the life of Emirati women that could protect or increase their chances of getting heart disease?

Does ‘hasad’ play a role in heart disease?

- How would people seek treatment if they believed it was ‘hasad’
- Would they seek medical treatment?

Black magic??

Do you believe we/you could be at risk for heart disease?

**Action**

What would you do if you thought you were having a heart attack?

Do you have a regular doctor you go to?

- Is a regular doctor important to you?
- Why do you prefer that particular doctor?
  - Female doctor- Do we have enough?
  - Nationality
  - Religion?
  - Language?

**Barriers**

Do you believe women delay seeking help for health problems?

- Explain

Is there anything that would prevent you from seeking health care?

- Do you believe that there is a difference between genders when it comes to willingness to seek treatment?
- Why do you believe so?

**Hesitancy to reveal they are ill (Cultural? Why?)**

- Cost of treatment in UAE?
- Quality of treatment in UAE?
- Insurance?
- Culture? Permission, family
- Transportation
- Self medication
- Alternative medicine

(Prompts to be used as required)

Would you prefer treatment abroad to UAE?

- Explain
- How do you feel about health care in UAE?

How do you feel about emergency health care in UAE?

How aware do you believe Emirati women are of heart disease risks and symptoms?

- How can we increase awareness on heart disease in women?
- Who has an important role to play?

Do you believe awareness on heart disease differs in different emirates?

- Explain
- Age groups
- Educational status
